# Evaluating the socio-demographic, economic and clinical (SDEC) factors on health related quality of life (HRQoL) of hypertensive patients using EQ-5D-5L scoring algorithm

**DOI:** 10.1371/journal.pone.0270587

**Published:** 2022-06-30

**Authors:** Nousheen Aslam, Muhammad Harris Shoaib, Rabia Bushra, Saima Asif, Yusra Shafique

**Affiliations:** 1 Department of Pharmaceutics, Faculty of Pharmacy and Pharmaceutical Sciences, University of Karachi, Karachi, Sindh, Pakistan; 2 Department of Pharmaceutics, Faculty of Pharmacy, Dow University of Health Sciences, Karachi, Sindh, Pakistan; 3 Faculty of Pharmacy, Jinnah University for Women, Karachi, Sindh, Pakistan; 4 Institute of Pharmaceutical Sciences, Jinnah Sindh Medical University, Karachi, Sindh, Pakistan; Lahore Pharmacy College, Punjab, Pakistan, PAKISTAN

## Abstract

This study was conducted to determine the various socio-demographic, economic, and clinical variables (SDECVs) which influence the health-related quality of life (HRQoL) of hypertensive patients. Three hundred and fifty hypertensive patients participated in this study through a structured questionnaire and EQ 5D 5L. 211(60.28%) participants had stage 1, and 139 (39.7%) had stage 2 hypertension. No participants reported severe problems in any domain on EQ 5D 5L. Generalize Linear Model (GLM) was used to assess the association between HRQoL and SDECVs. The mean utility and VAS score was 0.64 (±0.15) and 63.17 (±11.01) respectively. The participants of the stage 1 hypertension group had a significantly better score on each domain of EQ 5D 5L as compared to stage 1 (0.027, 0.010, 0.00, 0.00, 0.048). No participant in either group reported extreme problems in any domain. Among socio-demographic factors, the males, non-smokers, income sharing, and healthy normal hypertensive patients had better HRQoL (0.009, 0.016, 0.019, and 0.003). A lower cost of treatment was also associated with better HRQoL (0.017). Among clinical variables, stage 1 hypertension had better HRQoL than stage 2(0.035). The number of prescribed antihypertensive drugs had no effect on the quality of life (0.253), however, the non-pharmacologic interventions such as reduction in salt and oil consumption (0.035), reduction in beverages consumption (0.0014) and increased water intake (0.010) had resulted in better QoL. The patients who reported dizziness had poor HRQoL while patients who had cardiac problems and diabetes reported a significantly lower EQ-VAS score. The effect of gender on the HRQoL of hypertensive patients who had comorbid conditions was significant in the case of renal, respiratory, visual problems, and dizziness where females had a lesser utility score than males. The study reports on significant determinants which should be taken into account in an attempt to improve the health-related quality of life of hypertensive patients.

## Introduction

Hypertension and its associated complications are major global health concerns specifically for the elderly. Hypertension is a major mortality risk factor for many developing countries, including Pakistan. The disease not only produces a high economic burden on the country [1–3] it also affects the physical, social and mental well-being of hypertensive patients [4]. Moreover, it has been documented that reduced mental and physical health contributes to an increase in disease burden and a decline in life [5]. Therefore, assessment of health-related quality of life and its determinants may help decision-makers to improve policies for hypertension control, prevention, and treatment of hypertension in Pakistan [4].

Euro QoL 5D 5L is a generic preference-based HRQoL instrument that can help in deciding the most appropriate pharmacotherapy plan for suffering patients. EQ 5D 5L is reported to have reduced ceiling and floor effect, which results in better reliability of the instrument and discrimination between various levels of health, and it has been used successfully in HRQoL assessment in various diseases, including hypertension [6–9].

High blood pressure has been identified as a serious concern for health in developing countries still these countries have a scarcity of available data on the hypertension profile. Pakistan is no exception. There is not any nationally representative study on hypertension prevalence, disease burden, and health-related quality of life. Therefore, this comprehensive study aimed to examine the health-related quality of life of hypertensive patients in Karachi and its socio-demographic, economic and clinical determinants (SDECD) using a pre-validated EQ 5D 5L generic instrument. This is the first study of its type to determine various independent variables that influence health-related quality of life of hypertensive patients using EQ 5D 5L in this region, especially in Karachi, one of the most populous metropolises in the world.

## Methods

### Study design and ethics approval

It was a prevalence-based cross-sectional study that was approved by the “Advanced Studies and Research Board” (BASR) of the University of Karachi, Pakistan, and registered with the Euro QoL group. The study and its protocols were also reviewed and approved by the Institutional Bioethics Committee, University of Karachi (IBCPH 25-B-17).

### Sample size and inclusion/exclusion criteria

The sample size was calculated by Raosoft Calculator using 95% CI, 5% margin of error and 50% response distribution [10]. A total of three hundred and fifty (350) complete responses were collected after complying with inclusion criteria which included hypertensive patients ≥ 30 years of age, taking anti-hypertensive treatment for at least one year, could out rightly read, write and speak Urdu (the native language of Pakistan) and had knowledge about their own health status, disease condition, and their treatment profile. The participant’s consent was taken before they responded to the questionnaire.

### Sample collection

The data was collected from the out-patient clinics in least and vulnerable poor districts of Karachi, which cover 80% of the city’s health care services [11] through a Multicenter Data Collection (MDC) strategy using a stratified random sampling technique. Socio-demographic, economic and clinical information was collected through a structured questionnaire as Patient-Reported Outcome Measure (PROM), and the information about health related quality of life was obtained through EQ 5D 5L instrument. The validated Urdu version of E 5D 5L was used for this study.

### Classification of hypertension

High blood pressure was classified as Stage 1(90–99 to 140-159mmHg) and Stage 2 hypertension (≥100 to ≥160mm Hg) according to the guidelines provided by “The Pakistan Hypertension League (PHL) and the joint Navigation Committee (JNC) report on Prevention, Detection, Evaluation and Treatment of Hypertension” [12].

### Health-related quality of life (instrument)

Health-related quality of life (HRQoL) of hypertensive patients was calculated through “Euro QoL 5D 5L”. The study participants recorded their responses on the descriptive part and visual analogue scale. The descriptive component asks the patient to select one of the five levels in each of the five dimensions namely Mobility, Self-care, Usual Activity, Pain/Discomfort, Anxiety/Depression (hereafter M, SC, UA, PD, AD) as the best representation of their health state. These five levels are no problem (level = 1), slight problem (level = 2), moderate problem (level = 3), severe problem (level = 4), extreme problem/unable to do (level = 5). Thus, there are 3125 possible health states on EQ5D 5L. A health state “11111” means a perfect health and “55555” means worst health. The study participants also selected one point from the 20 cm long visual analogue scale (VAS) to report their perceived health state. The two endpoints of this scale are called “best imaginable health” and “Worst imaginable health”. The scale has 10 readings from “0” to “100” and the study participants are asked to rate their current health states on this scale [13]. The index value was calculated using a UK value set available for the EQ-5D 5L [4]. The HRQoL was calculated as a dependent variable to the various socio-demographic, economic and clinical variable (SDECV).

### Data analysis

Data was analyzed through descriptive (frequencies, percentages, standard deviations) and inferential statistics using Statistical Package for Social Science (SPSS V.23 Inc.). General Linear Model (GLM), a common method for response modeling problems [14, 15] was used to determine the effect of SDECV on the physical, mental and social domains of EQ 5D 5L.

## Results and discussion

High blood pressure has been identified as a serious concern for health in developing countries still, there is a scarcity of available data on the hypertension profile in these countries. Pakistan is no exception. There is also no nationally representative study on hypertension prevalence, disease burden, and health-related quality of life [3]. Therefore, this study determined various independent variables which influence the health-related quality of life of hypertensive patients using EQ 5D 5L.

The socio-demographic characteristics (Table 1) are presented as stage 1 (211, 60.28%) and stage 2(139, 39.7%) hypertension. The mean age of hypertensive patients was less than 55 years in both groups, and a majority of the patients had been suffering from hypertension for 6–10 years (7.85±3.5). However, the study could not establish an association between the stage and duration of hypertension (p = 0.367) which showed that blood pressure might rise silently until it is detected and monitored. The number of female hypertensive patients was more than males in both groups, although this difference was insignificant (*p* = 0.78) which is in contrast with other studies [3] (4,; Shah et al in 2018. There was no significant difference between marital status, occupation, family members, family income, and smoking habits between the two groups (*p* = 0.96, 0.78, 0.604, 0.759, and 0.196). The education status and body mass index were significantly less in stage 1 as compared to stage 2 (*p* = 0.026; 0.001).

**Table 1:** Socio-demographic characteristics.

Codes*	Characteristics	Stage 1(N = 211, 60.28%)	Stage 2 (N = 139, 39.7%)
	**AGE (Mean Years)**	53.39 ±10.32	54.69 ± 11.38
***1***	*30–45 years*	46 (21.8%)	21(15.6%)
***2***	*46–60 years*	112 (53.1%)	75 (38.8%)
***3***	*61–75 years*	46 (21.8%)	35 (15.7%)
***4***	*76 years and above*	7 (3.31%)	68 (2.4%)
	**SEX**		
***1***	*Male*	97 (45.97%)	66 (47.48%)
***2***	*Female*	114 (54.02%)	73 (52.5%)
***1***	**Systolic BP**	131.13(±9.59)	165.79 (±16.65)
***2***	**Diastolic BP**	98.85 (±16.46)	102.23(±14.9)
	**BODY MASS INDEX (BMI)**		
***1***	*Healthy Normal(18*.*5–24*.*9)*	129 (61.1%)	24 (17.2%)
***2***	*Overweight(25–29*.*9)*	64 (30.3%)	83 (59.7%)
***3***	*Obese (>30)*	18 (8.5%)	32 (23.07%)
	**MARITAL STATUS**		
***1***	*Married*	201(95.2%)	130 (93.5%)
***2***	*Widow/divorced*	4 (1.89%)	3 (2.15%)
***3***	*Single/Never married*	6 (2.84%)	3 (2.15%)
	**EDUCATION**		
***1***	*Secondary School*	99 (46.91%)	50 (35.97%)
***2***	*Higher Secondary School*	49 (23.2%)	28 (20.14%)
***3***	*Graduate*	40 (18.95%)	37 (26.6%)
***4***	*Master/Postgraduate*	23 (10.9%)	24 (17.2%)
	**OCCUPATION STATUS**		
***1***	*Job/Earning through any mode*	144 (68.24%)	100 (71.9%)
	_Household Head	100 (69.4%)	78 (78%)
	_Income Sharing	44 (30.5%)	22 (15.82%)
***2***	*Not Earning/Dependent*	67 (31.75%)	39 (28.05%)
	**FAMILY MEMBERS**	7 ±2.2	6.8 ±2
***1***	*2–5*	4 (21.32%)	35(25.17%)
***2***	*6–10*	154 (72.98%)	100 (71.94%)
***3***	*11 and above*	12 (5.68%)	4 (2.87%)
	**DURATION OF HYPERTENSION (years)**		
***1***	*1–5*	63 (29.85%)	46 (33%)
***2***	*6–10*	86 (40.75%)	47 (33.81%)
***3***	*11–15*	62 (29.38%)	46 (33%)
	**ALCOHOL CONSUMPTION**		
***1***	*Yes*	0	0
***2***	*No*	211(100%)	139 (100%)
	**SMOKING/TOBACCO CONSUMPTION**		
***1***	*Yes*	15 (7.10%)	7 (5.03%)
***2***	*No*	174(82.46%)	94(67.62%)

The percent response to the descriptive part of EQ 5D 5L is present in Table 2. No participants reported “11111” and “55555” states. There was no response for the extreme problem in any domain. The GLM (Table 3) presented the linear regression between five levels of each domains of EQ 5D questionnaire and different socio-demographic, economic, and clinical variables. A significant *p* value means that the problem on this domain increased with the variable. The codes provided in Table 1 can be used to interpret the GLM. The problems in mobility, self-care, and usual activities significantly increased with increased family income and increased education and marital status, respectively. The issues in PD and AD were more in females and increased with an increase in family income, respectively. Non-smokers showed more problems in PD. The other socio-demographic characteristics like occupation and marital status, BMI and family members did not show any linear association with any of these five domains.

**Table 2:** Percent response to each level of five dimensions on EQ-5D 5L.

Dimensions of EQ-5D	Level	Level Description	% Response		
			Stage 1 N (%)	Stage 2 N (%)	Difference *p-value*
**Mobility**	**1**	**No problem**	111 (52.6)	59(42.44)	
	**2**	**Some problem**	7(3.31)	69(49.64)	0.027
	**3**	**Moderate problem**	84(39.81)	-	
	**4**	**Severe problem**	9(4.26)	11(7.9)	
	**5**	**Extreme problem/unable to do**	-	-	
**Self-Care**	**1**	**No problem**	107(50.7)	53(38.12)	
	**2**	**Some problem**	11(5.21)	77(55.39)	0.010
	**3**	**Moderate problem**	90(42.65)	5(3.59)	
	**4**	**Severe problem**	3(1.42)	4(2.87)	
	**5**	**Extreme problem/unable to do**	-	-	
**Usual Activities**	**1**	**No problem**	72(34.12)	48(34.5)	
	**2**	**Some problem**	7(3.31)	64(46)	0.00
	**3**	**Moderate problem**	127(60.18)	15(10.79)	
	**4**	**Severe problem**	5(2.36)	12(8.63)	
	**5**	**Extreme problem/unable to do**	-	-	
**Pain/Discomfort**	**1**	**No problem**	64(30.33)	33(23.7)	
	**2**	**Some problem**	9(4.26)	64(46)	0.00
	**3**	**Moderate problem**	118(55.9)	15(10.79)	
	**4**	**Severe problem**	20(9.4)	12(8.63)	
	**5**	**Extreme problem/unable to do**	-	-	
**Anxiety/ Depression**	**1**	**No problem**	92(43.6)	55(39.56)	
	**2**	**Some problem**	9(4.26)	55(29.56)	0.048
	**3**	**Moderate problem**	94(44.54)	15(10.79)	
	**4**	**Severe problem**	16(7.58)	14(10)	
	**5**	**Extreme problem/unable to do**	**-**	**-**	

p-value is significant at <0.05.

**Table 3:** Generalize linear model (GLM) to predict the effect of SDEC variables on EQ 5D 5L domains.

	Mobility	Self-care	Usual activities	Pain/ Discomfort	Anxiety/ Depression
**Socio-demographic and Economic Variables**					
**Gender**	0.940	0.361	0.524	***0*.*046*** *	***0*.*047*** *
**Marital Status**	0.710	0.109	***0*.*049***	0.082	0.549
**Education**	0.252	***0*.*044*** *	0.862	0.397	0.904
**Smoking**	0.553	0.518	0.353	***0*.*048*** *	0.359
**family members**	0.054	0.134	0.052	0.146	0.266
**Family income**	***0*.*049***	0.451	0.085	***0*.*044***	***0*.*027***
**Clinical Variables**					
**BP**	***0*.*027*** *	***0*.*01*** *	***0*.*01*** *	***0*.*00*** *	***0*.*048*** *
**number of drugs**	0.077	***0*.*001***	0.947	0.943	0.644
**co-morbid conditions**	0.575	***0*.*007***	0.268	***0*.*001***	0.306
**Renal Problems**	0.995	0.850	***0*.*005*** *	0.805	0.573
**Cardiac problem**	0.272	0.256	***0*.*003*** *	0.288	0.846
**Dizziness**	0.560	***0*.*000*** *	0.861	***0*.*000*** *	***0*.*000*** *
**Diabetes**	***0*.*004*** *	***0*.*013*** *	***0*.*006*** *	***0*.*010*** *	0.074

The table represents only those SDECV who have significant association with at least one of the five domains of EQ 5D 5L.

*significant values

The socio-demographic and economic variables which affected the HRQoL of hypertensive patients are present in Table 4. Male participants as compared to female, non-smokers as compared to smokers, income sharing as compared to household heads and dependents, healthy normal participants as compared to overweight and obese hypertensive patients had better Health related quality of life (*p* = 0.009, 0.016, 0.027, 0.019, 0.003). The hypertensive patients whose annual cost of hypertension was low had a better HRQoL than the patients who spent more for the treatment of hypertension (*p* = 0.017).

**Table 4:** Effect of socio-demographic, economic and clinical variables on health related quality of life.

**Socio-demographic and Economic Variables**						
			**EQ 5D Index value**		**EQ VAS Score**	
		**N**	**(Mean ± SD)**	***p* value**	**(Mean ± SD)**	***p* value**
**Gender** *	Male	163	0.659(±0.13)	***0*.*009***	62.84(±11.7)	***0*.*017***
	Female	187	0.616(±0.18)		60.5(±11.4)	
**Smoking** *	Yes	22	0.6003(0.14)	0.113	57.72(11.1)	***0*.*016***
	No	268	0.654(0.15)		63.56(10.01)	
**Family members** **	2–5	81	.6592(.14833	0.538	63.5556(12.4)	0.622
	6–10	255	.6471(.15506		63.2157(10.7)	
	11–20	14	.6191(.09335		60.(8.98)	
**Occupational Status** **	household heads	178	0.63 (0.16)	***0*.*019***	62(10.9)	***0*.*019***
	income sharing	66	0.69 (0.12)		65.3(10.6)	
	Not earning/dependent	106	0.65(0.14)		63.7(11.4)	
**BMI** **	Healthy Normal	285	0.66(0.14)	***0*.*003***	63.16 (11.1)	0.503
	Overweight	58	0.618(0.19)		63.6(10.9)	
	Obese	6	0.64(0.139)		57.5(10.8)	
**Income (PKR)** **	10–50,000	263	0.62(0.10)	***0*.*027***	63.72 (6.49)	0.371
	51–100,000	69	0.631 (0.17)		64.27(4.61)	
	Above 100,000	18	0.64(0.13)		64.95(3.0)	
**Annual Cost of hypertension**	100,000–200,000	166	0.66(0.08)	***0*.*017***	61.5(10.2)	0.750
**Clinical Variables**						
**BP** *	Stage 1	211	0.649 (±0.1)	***0*.*035***	64 (±10.6)	***0*.*03***
	Stage 2	139	0.647 (±0.16)		61(±11.6)	
	Above 200,000	184	0.648 (0.15)		63.5(11.1)	
**Reduction in salt and oil consumption** *	Yes	278	0.659(±0.13)	***0*.*035***	62.84(±11.7)	***0*.*051***
	No	72	0.61(0.17)		60.3(10.35)	
			**EQ 5D Index Value**		**EQ VAS Value**	
		**N**	**(Mean ± SD)**	**p value**	**(Mean ± SD)**	**p value**
**Reduction in beverages consumption** *	Yes	281	0.64(0.15)	***0*.*0014***	64(11.1)	0.26
	No	68	0.67(0.12)		658(11)0.260	
**Increased Water intake** *	Yes	295	0.65(0.15)	***0*.*010***	63.8(11.2)	0.686
	No	55	0.64(0.12)		59.6(9.66)	
**Increased exercise** *	Yes	222	0.65(0.14)	0.71	62.9(11.1)	0.9
	No	127	0.648(0.15)		63.4(11.1)	
**Antihypertensive drugs (Numbers)** **	1	67	0.65(0.15)	0.253	63.95(10.4)	0.056
	2	199	0.656(0.146)		63.99(11.2)	
	3	83	0.648(0.151)		60.84(11.7)	
**Co-morbid conditions (Numbers)** **	1	16	0.6(0.21)	***0*.*004***	68.13(11.6)	0.067
	2	40	0.72(0.11)		65.2(11)	
	3	293	0.64(0.15)		62.6(11)	
**Renal Problems** *	Yes	292	0.655(0.14)	0.182	62.8(11.1)	0.124
	No	57	0.62(0.18)		64.9(10.75)	
**Respiratory Problem** *	Yes	294	0.652(0.14)	0.150	62.7(11.1)	0.339
	No	55	0.633(0.175)		65(10.8)	
**Visual Problem** *	Yes	147	0.64(0.147)	0.773	63.5(10.4)	0.608
	No	203	0.65 (0.145)		62.9(11.6)	
**Cardiac problem** *	Yes	115	0.64 (0.165)	0.227	61.1(11.38)	***0*.*014***
	No	235	0.66 (0.14)		64.18(10.8)	
**Dizziness** *	Yes	250	0.61(0.15)	***0*.*000***	63.0	0.722
	No	100	0.71(0.07)		63.5	
**Diabetes** *	Yes	187	0.64(0.159)	0.968	62(11.3)	***0*.*042***
	No	162	0.648(0.142)		64.4(10.6)	

*Independent Sample t-test;

**ANOVA, *p-value is significant at <0*.*05*

**Fig 1:**
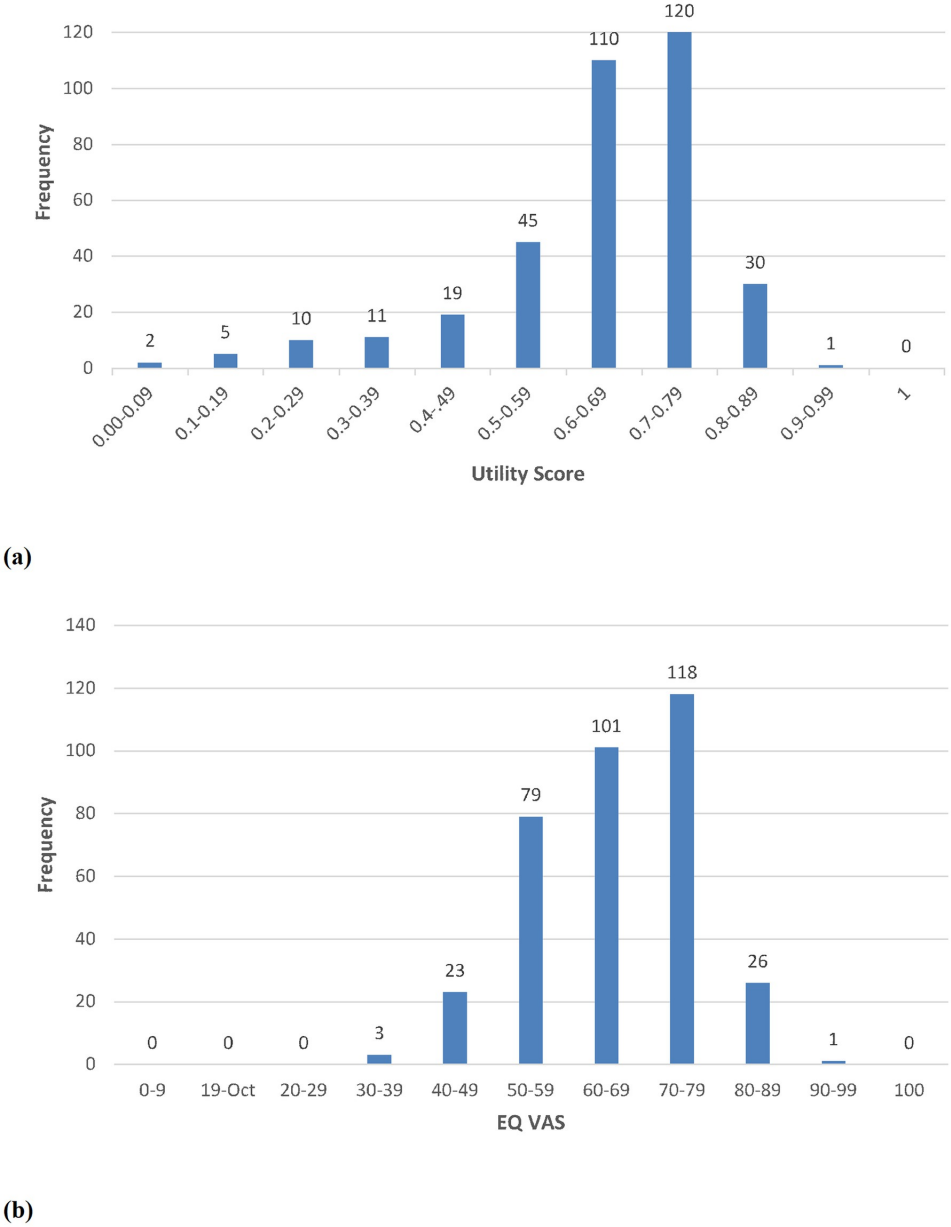
(a) Utility Scores of hypertensive patients (N = 350) (b) EQ Visual Analogue Scale (EQ VAS) of hypertensive patients (N = 350).

The mean utility and VAS score was 0.64 (±0.15) and 63.17 (±11.01) respectively (Fig 1a and 1b). Age, gender, and income are those socio-demographic variants that are closely associated with hypertension in Pakistan [16–18]. Our study did not find age as a predictor for HRQoL of hypertensive patients (*p* = 0.37); however gender, smoking habit and income had significant effects on health related quality of life. Male patients had a better HRQoL than females. This is in agreement with other previous studies [4, 18]. Smoking by hypertensive patients is a serious risk factor as it can result in malignant and renovascular hypertension due to accelerated atherosclerosis. Many studies have previously reported a positive correlation between smoking and the development of hypertension in Pakistan [19]. Still, none had reported a significant effect of smoking on HRQoL of hypertensive patients as it is reported in this study.

Body mass index did not show a direct effect on the health related quality of life (*p* = 0.236). BMI has a significant positive correlation with blood pressure (*p* = 0.001); therefore, it may affect HRQoL indirectly. Similarly, females had a higher BMI (p = 0.008) than males, which may be a reason for the lower HRQoL of females [20–22].

Income was the only economic factor that significantly affected the HRQoL of hypertensive patients in this study. Respondents with high income reported a better HRQoL on the EQ value index. However, there was no association between income and blood pressure (*p* = 0.76). At the same time, the high cost of hypertension treatment was significantly associated with a lower HRQoL. However, there was a linear significant correlation between income and total cost of treatment (*p* = 0.015) which shows that high income could help the hypertensive patients to spend more on their treatment, as supported by previous studies [23]. Although, this association did not help to build a significant positive association between spending on hypertension treatment and HRQoL. It cannot be concluded that a higher cost of treatment can result in better health care and health-related quality of life of patients. The cost of treatment was calculated from a patient’s perspective [2]. Therefore, this finding urges the decision-makers in Pakistan for a closer look at out-of-pocket expenditure on hypertension treatment and demands for a change in policy for the provision of cost-effective treatment options for hypertensive patients. We can also assume that this is another reason for poor BP control in Pakistan, where prevalence of hypertension is 33%. 50% of hypertensive patients are diagnosed, and half of these diagnosed patients receive treatment. Only 12.5% of these patients have controlled BP. A high cost of treatment is also responsible for low medication adherence and medication adherence is significantly associated with HRQoL of hypertensive patients in Pakistan [19, 24].

It was also found that the hypertensive household heads had a significantly poor HRQoL when compared to the other members. No previous study was found showing this relationship, and it can be figured out in future studies.

The clinical variables included blood pressure, duration of hypertension, pharmacological (anti-hypertensive drugs), non-pharmacological (life style modifications) treatments and co-morbid conditions. As indicated before in this section that the level of problems linearly and significantly increased with the increase in blood pressure, the utility and EQ VAS score of stage 2 hypertensive patients were also significantly lower than stage 1.

The GLM also gives an insight that nonusers of some anti-hypertensive agents showed a significant linear relationship with severity of problems in different domains However, there was no significant difference in the utility and EQ-VAS score between users and non-users of anti-hypertensive drugs. The number of drugs that were being consumed by the hypertensive patients was linearly and significantly associated with problems in self-care. Different drugs showed a positive significant association with various domains such as angiotensin-converting enzyme inhibitors (SC), angiotensin receptor blockers (M), beta-blockers (M), and calcium channel blockers (AD), diuretics (AD), and alpha-blockers (AD). However, the number of drugs did not cause any difference in the health-related quality of life.

Non-pharmacological treatment did not show any linear relationship with the level of problems on GLM. Although all those participants who reduced the salt and oil consumption in their diet, reduced the use of beverages and increased water consumption had significantly better HRQoL than those who did not opt for these alterations (*p* = 0.035, 0.004, 0.010, respectively). Exercise did not show any significant difference in the HRQoL of participants.

The participants also reported some co-morbid conditions such as renal, respiratory, visual, cardiac problems, diabetes and also reported dizziness with the switch in their blood pressure as reported in other studies also [25, 26]. The hypertensive patients who had renal and cardiac problems showed increased problems in usual activities. The participants who suffered from dizziness reported significant issues with self-care, pain/discomfort, and anxiety depression. The hypertensive diabetic patients showed significant issues in all domains except anxiety/depression. However, the hypertensive patients who did not have diabetes and cardiac problems reported significantly higher EQ VAS scores. Those who did not suffer from dizziness had a significantly better utility score. The hypertensive patients who had 2 co-morbid conditions with hypertension had better utility scores than the participants with 1 and 3 co-morbid conditions. Male hypertensive patients who had renal, respiratory, visual and cardiac problems had significantly better health related quality of life than respective female hypertensive patients (Table 5). There was no significant difference in the HRQoL of males and female hypertensive patients with diabetes and dizziness.

**Table 5:** Effect of gender on HRQoL of hypertensive patients with different Co-morbid conditions.

Effect of Gender on HRQoL of hypertensive patients with different Co-morbid conditions						
Comorbid Condition	Gender	N	EQ 5D Index value		EQ VAS Score	
			(Mean ± SD)	*p* value	(Mean ± SD)	*p* value
**Renal Problem**	Male	123	0.68(0.13)	***0*.*013***	64.5 (11.0)	***0*.*02***
	Females	169	0.63(0.162)		61(11.0)	
**Respiratory Problem**	Male	125	0.674(0.12)	***0*.*006***	64 (11.01)	***0*.*020***
	Female	169	0.625(0.166)		61 (11.01)	
**Visual Problem**	Male	74	0.67(0.133)	***0*.*039***	63.45(10.1)	0.947
	Female	73	0.622(0.15)		63.58(10.74)	
**Cardiac Problem**	Male	96	0.70(0.092)	***0*.*007***	60.52(10.65)	0.807
	Female	19	0.623(0.174)		61.19(11.57)	
**Dizziness**	Male	128	0.625 (0.13)	0.149	64.4 (10.64)	***0*.*048***
	Female	122	0.60(0.17)		61.7 (10.80)	
**Diabetes**	Male	49	0.66(0.13)	0.709	63.5(11.27)	0.766
	Female	138	0.66(0.144)		62.95(10.74)	

p-value is significant at <0.05

It is well documented that changes in lifestyle positively aids in controlling blood pressure and attaining a better health related quality of life [27] which was well proved in this study. The participants who had reduced their dietary salt intake, reduced the consumption of oil and beverages like carbonated drinks, and increased the water intake had significantly better HRQoL than hypertensive patients who did not embrace these modifications in their lifestyle.

The number of drugs increased with the increase in blood pressure (*p* = 0.001), but the health-related quality of life was not improved with the addition of anti-hypertensive drugs in the regimen. It was found that the participants who were taking 2 anti-hypertensive drugs had better HRQoL than those who were taking 3 drugs (*p* = 0.02). However, there was no overall difference in the HRQoL of hypertensive patients taking anti-hypertensive drugs to control their blood pressure. Some previous studies have reported that an increase in the number of antihypertensive drugs reduces the patient adherence to the therapy, which can be a reason for poor HRQoL of hypertensive patients [28–30]. However, there is no direct relationship between the poor quality of life and the use of antihypertensive drugs [31, 32].

The goal of antihypertensive treatment is to reduce or control blood pressure, which can be linked with an improved quality of life. Since this study did not focus on treatment adherence, we cannot establish a direct relation between HRQoL and the use of medications to control BP. However, a linear correlation resulted in significant inverse relation between EQ VAS and the number of anti-hypertensive drugs (r = -0.83, *p* = 0.039), which may lead to a further investigation for the association between a number of anti-hypertensive medications and the health related quality of life of hypertensive patients.

Co-morbid clinical conditions have a negative effect on the quality of life of hypertensive patients [33, 34]. The co-morbid conditions increased significantly with the increase in the duration of hypertension (r = 0.129, *p* = 0.016) and blood pressure (r = 0.145, *p* = 0.001), leading to the poor health-related quality of life of hypertensive patients as reported by other studies. There is no available national study to support this finding but some international studies have reported that men had better HRQoL with comorbidities than women [35].

## Conclusion

High blood pressure has been a concern for developing countries and the scarcity of data about hypertension-associated health-related quality of life and its determinants makes the situation worse. The present study has identified various significant and controllable factors which can serve as focus of attention for healthcare practitioners and policy makers. Such as gender, lifestyle, pharmacologic and non-pharmacologic interventions, blood pressure itself, and the associated clinical conditions. The income of individuals and the cost of hypertension treatment has also been determining factors for the HRQoL of hypertensive patients. The poor quality of life of individuals has a negative impact on overall productivity of the society. The control of hypertension and its effects on the quality of life of patients is crucial however, the authors suggest that gender-based health care need assessment can be a useful tool to control the poor health-related quality of life. Lifestyle interventions such as awareness about non-pharmacologic measures and smoking cessation campaigns can be initiated by both public and private health care organizations to educate hypertensive patients to manage their blood pressure through these life style modifications and live a better quality of life. The policymakers should identify the areas where cost containment is necessary to control the economic burden of hypertension treatment from the individual perspective which may result in better quality of life. The financial support by government and private health care organizations to less privileged individuals can also help in improving their quality of life.

### Limitations of the study

Although this multicenter study has yielded important information about the health-related quality of life of hypertensive patients from the metropolitan city of Pakistan using validated EQ 5D 5L, the study has some limitations which can be addressed in future studies. Such as the inclusion of the regional language speakers, gathering data on a national scale (both rural and urban areas), and the role of health care organizations and policymakers in the control and management of hypertension and improvement of the quality of life of hypertensive patients could not be addressed in this study.

## Supporting information

S1 File Raw data excel file for Tables 1–6. (XLSX)
